# Use of an Electronic Medication Management Support System in Patients with Polypharmacy in General Practice: A Quantitative Process Evaluation of the AdAM Trial

**DOI:** 10.3390/ph15060759

**Published:** 2022-06-17

**Authors:** Robin Brünn, Dorothea Lemke, Jale Basten, Petra Kellermann-Mühlhoff, Juliane Köberlein-Neu, Christiane Muth, Marjan van den Akker, on behalf of the AdAM Study Group

**Affiliations:** 1 Institute of General Practice, Goethe University, 60590 Frankfurt am Main, Germany; lemke@allgemeinmedizin.uni-frankfurt.de (D.L.); christiane.muth@uni-bielefeld.de (C.M.); m.vandenakker@allgemeinmedizin.uni-frankfurt.de (M.v.d.A.); 2 Department of Medical Informatics, Biometry and Epidemiology, Ruhr University, 44789 Bochum, Germany; basten@amib.ruhr-uni-bochum.de; 3 BARMER, Statutory Health Insurance, 42285 Wuppertal, Germany; petra.kellermann-muehlhoff@barmer.de; 4 Center for Health Economics and Health Services Research, Schumpeter School of Business and Economics, University of Wuppertal, 42119 Wuppertal, Germany; koeberlein@wiwi.uni-wuppertal.de; 5 Department of General Practice and Family Medicine, Medical Faculty East-Westphalia, University of Bielefeld, 33615 Bielefeld, Germany; 6 Department of Family Medicine, Care and Public Health Research Institute, Maastricht University, 6200 Maastricht, The Netherlands; 7 Department of Public Health and Primary Care, Academic Centre of General Practice, KU Leuven, 3000 Leuven, Belgium

**Keywords:** polypharmacy, medication review, digital decision-support, health services research, general practice, process evaluation

## Abstract

Polypharmacy is associated with a risk of negative health outcomes. Potentially inappropriate medications, interactions resulting from contradicting medical guidelines, and inappropriate monitoring, all increase the risk. This process evaluation (PE) of the AdAM study investigates implementation and use of a computerized decision-support system (CDSS). The CDSS analyzes medication appropriateness by including claims data, and hence provides general practitioners (GPs) with full access to patients’ medical treatments. We based our PE on pseudonymized logbook entries into the CDSS and used the four dimensions of the Medical Research Council PE framework. Reach, which examines the extent to which the intended study population was included, and Dose, Fidelity, and Tailoring, which examine how the software was actually used by GPs. The PE was explorative and descriptive. Study participants were representative of the target population, except for patients receiving a high level of nursing care, as they were treated less frequently. GPs identified and corrected inappropriate prescriptions flagged by the CDSS. The frequency and intensity of interventions documented in the form of logbook entries lagged behind expectations, raising questions about implementation barriers to the intervention and the limitations of the PE. Impossibility to connect the CDSS to GPs’ electronic medical records (EMR) of GPs due to technical conditions in the German healthcare system may have hindered the implementation of the intervention. Data logged in the CDSS may underestimate medication changes in patients, as documentation was voluntary and already included in EMR.

## 1. Introduction

Life expectancy around the world has risen as a result of improvements in the diagnosis and treatment of chronic and acute diseases, and better living conditions and hygiene [1]. Longer lives increase the likelihood of developing chronic diseases—if more than one disease occurs in one person at the same time—a condition known as multimorbidity [2]. As increasingly specialized clinical experts use increasingly complex pharmacotherapies to treat individual diseases, while insufficiently taking into account a patient’s multimorbidity, polypharmacy—usually defined as the concurrent intake of at least five different chronic medications [3]—is becoming ever more common [4,5]. There is growing awareness that polypharmacy should itself be treated as a risk factor, since the parallel treatment of different diseases with pharmacotherapy can have contradicting or reinforcing effects that are potentially life-threatening [6]. Polypharmacy is, for example, associated with higher rates of hospitalization [7,8,9] and death [9], as well as decreased quality of life and higher symptom burden [10].

The use of computerized decision-support systems (CDSS) to prevent or manage problematic polypharmacy has been evaluated in previous studies, and been shown to improve prescribing quality and reduce the prescription of potentially inappropriate medication [11,12]. However, the results have not always been significant [13], and their impact has frequently only been considered independently of patient-relevant outcomes. When they have been linked to patient-relevant outcomes, results have been inconsistent and have lacked robustness [14,15]. Since such interventions are generally complex, knowledge is lacking on what parameters lead to what outcomes, and on whether interventions have actually been implemented as intended. To gain a better understanding of the processes underlying complex interventions, it is recommended that they are accompanied by a preplanned process evaluation [16,17,18]. A comprehensive process evaluation is also very helpful when complex interventions do not show the anticipated effects. In these cases, the aim of process evaluations is to find the reason(s) for the observed lack of effectiveness. However, few trials follow this advice [19,20].

To circumvent these problems in the AdAM study, a process evaluation based on data logged by a CDSS named “eMMa” (a detailed explanation of the functioning can be found in the Methods section) was carried out with the aim of gaining an insight into how and why the AdAM intervention works the way it does. The study protocol has been published elsewhere a priori [21]. This paper presents the results of the process evaluation of the AdAM intervention, whereby the recommendations of the Medical Research Council (MRC) framework for process evaluations of complex interventions [18] helped us decide on which results to present and on how to structure our report. They also enabled us to provide a multidimensional view of the actual interventions. We ultimately settled on the following research questions: (1) How many and what were the typical characteristics of the GPs and patients that took part in the intervention (i.e., the Intervention “Reach”)? (2) What proportion of medication alerts were handled by GPs, and which were considered high priority (Intervention “Dose”, not to be confused with the dosage of a specific drug)? (3) What proportion of participants received the intervention as intended, thus increasing the likelihood of success (Intervention “Fidelity”)? (4) How did GPs integrate the intervention into their daily routines (Intervention “Tailoring”)?

## 2. Results

### 2.1. Intervention Reach

**Figure 1:**
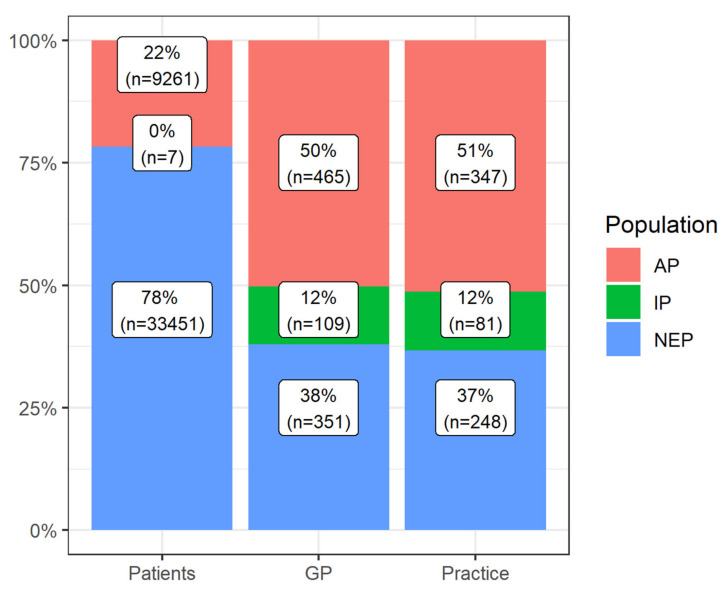
Distribution of patients, GPs, and practices in the defined populations (AP: active population, IP: inactive population, NEP: non-enrolled potentials).

The “Reach” intervention dimension refers to the participants that were actually reached by the intervention. Overall, 42,719 patients were considered potentially eligible in the main intention-to-treat analysis, of whom 9268 patients enrolled and 9261 (22%) showed up in the software (here called the “active population”: AP). The remaining 33,451 patients (78%) did not enroll in the study and made up the non-enrolled potentials (NEP), along with their respective GPs (*n* = 351, 34%) and practices (*n* = 248, 37%), if they agreed to participate in the trial (see Figure 1). Table 1 shows a comparison between the AP and NEP groups for patients. As only 7 of the enrolled patients did not appear in the software (“inactive population”: IP), statistical analysis was not feasible for them. Of 925 eligible GPs from 676 practices, 574 GPs (62%) from 428 practices (63%) agreed to participate in the AdAM study. Of these, 465 GPs (50%) from 347 practices (51%) were identified as having actively used the software program and thus comprised the AP. The remaining 109 GPs (12%) and 81 practices (12%) belonged to the IP. Since this analysis focuses on the data gathered from the CDSS, no comparison with potentially eligible GPs and practices in the study region could be conducted. Table 2 and Table 3 compare the AP to the IP and NEP for GPs and practices.

Compared to the NEP patient population, the AP patient group contains more men and is slightly older. The nursing care level is defined in German social security laws and specifies the need for nursing services and the welfare payment a patient is entitled to. A higher nursing care level indicates a greater need for nursing services and a higher welfare payment. With an increasing nursing care level, it was less likely that patients would receive the intervention.

On average, GPs that actively used the software were younger than those that had only inactive or non-enrolled patients. Group practices and practices that were randomized to the intervention group from the beginning were also more frequent users than practices that switched to the intervention group in later waves. No other characteristics had a significant impact on CDSS usage.

### 2.2. Intervention Dose

**Figure 2:**
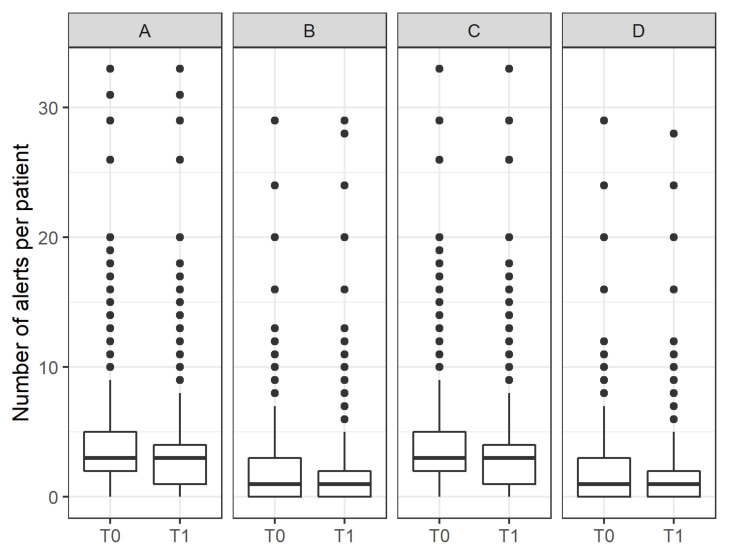
Box plot showing the number of alerts per patient at T_0_ and T_1_ (**A**), stratified according to the subgroups “severe alerts” (**B**), “completed anamnesis” (**C**), and a combination of both subgroups (**D**).

**Figure 3:**
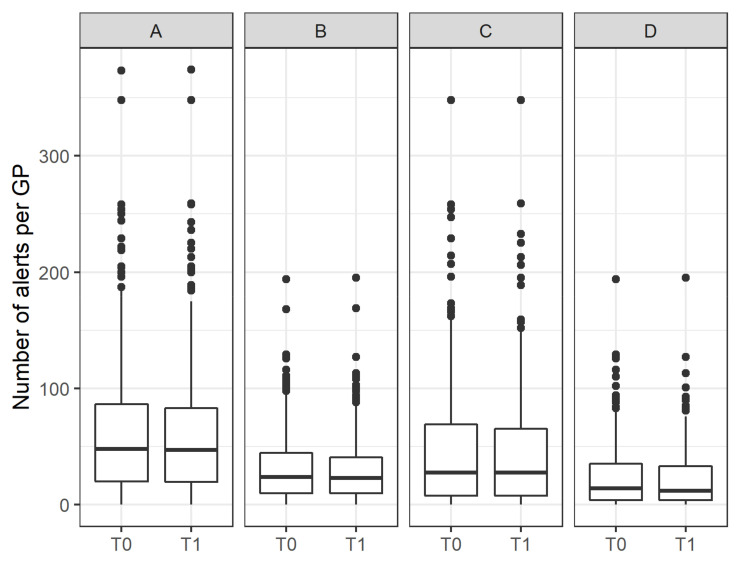
Box plot showing the number of alerts per GP at T_0_ and T_1_ (**A**), stratified according to the subgroups “severe alerts” (**B**), “completed anamnesis” (**C**) and a combination of both subgroups (**D**).

The “Dose” intervention dimension provides an insight into the extent to which the intervention was adopted and implemented, i.e., refers to the “dose” of the intervention the participants actually received. Figure 2 and Figure 3 show the distribution of the number of alerts per patient and GP, as tracked by eMMa before and after the intervention. Overall, the numbers remained constant, indicating no improvement in prescribed medications. An analysis of cases in which a complete anamnesis had been performed and confirmed by GPs, showed a modest reduction in the median number of alerts per patient.

**Figure 4:**
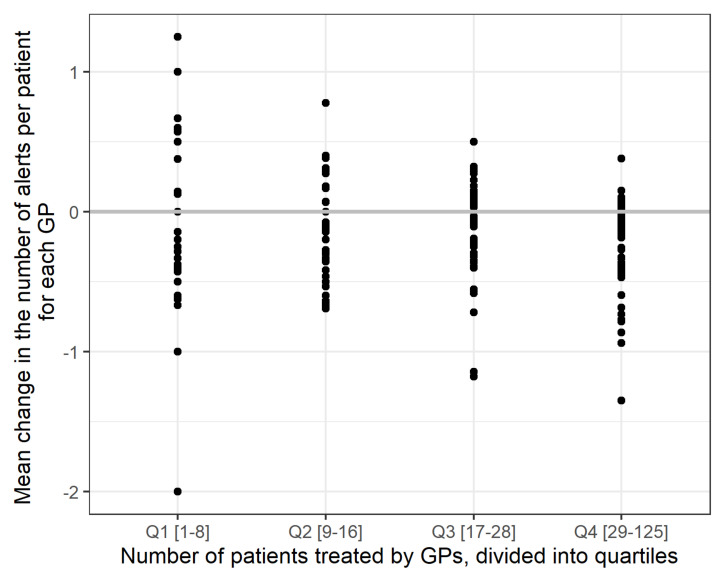
Average change in the number of alerts per GP, stratified according to the number of patients from the active study population treated by the GP (expressed in quartiles).

After adjusting the number of alerts per GP to take account of the number of treated patients (Figure 4), the alert count remained virtually constant (mostly a max. of +/− 1 alert per patient), but there is an inverse correlation between the overall number of patients treated by a GP and the change in the number of alerts.

Stratifying by alert category gives an insight into the kinds of potential inappropriateness that were assessed with a higher priority (Table 4). Since the software did not generate alerts for Dear Doctor Letters, no analysis was conducted in this category. The number of alerts warning of an inappropriate dosage or the unsuitability of a medication in view of a patient’s kidney function declined most frequently. GPs did not appear to pay much attention to alerts relating to potential allergies or duplicate prescriptions, and these actually increased.

The reduction was greatest when severe alerts and patients that had received a complete anamnesis were the only groups taken into consideration. Potential drug–drug interactions are reacted to much more frequently when the CDSS rates them as “severity level 1”.

Poisson regression analysis resulted in the incidence rate ratios shown in Table 5. Sensitivity analysis for justified alerts is depicted in Table 6. Significant differences in incidence rate ratios are shown in bold.

Overall, there were only a few significant reductions in alerts, and these were solely in the dosage category (Table 5). However, when alerts flagged as “justified” were left out of the analysis, the picture changed, and a significant reduction in dosage alerts could be detected in all subgroups (Table 6). The same is true for almost all kidney alert subgroups. Point estimates for severity level 1 alerts are equal or lower than those of alerts overall, but the significance of the reduction was limited by low case numbers.

### 2.3. Intervention Fidelity

The “Fidelity” intervention dimension evaluates how frequently the intervention was implemented in such a way that it was actually possible to achieve the aim of the intervention. Table 7 shows how many patients had severe alerts at T_0_ and how many of these cases were satisfactorily dealt with according to the criteria defined in the methods section (confirmed completed anamnesis and zero unjustified alerts of severity level 1 at T_1_). The alerts were stratified by category. On a GP level, no participant fulfilled the Fidelity criteria in any given category for all their patients.

As in the case of the Dose dimension findings, duplicate prescriptions and allergies received little attention in comparison to the other categories.

As far as the more highly prioritized categories are concerned, the GPs acknowledged and dealt with all the severe alerts in fewer than 30% of patients, indicating that the intervention goal was only moderately fulfilled.

**Figure 5:**
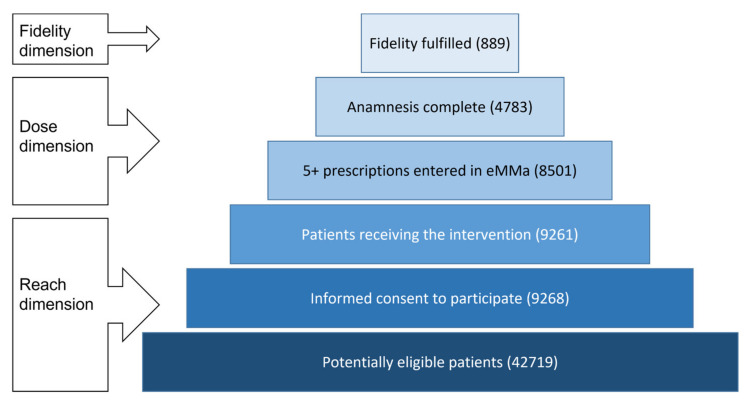
Steps to be climbed in order to achieve Intervention Fidelity. The left-hand legend shows the dimensions impacted by the steps.

Summarizing over all categories, severe alerts were only fully resolved or justified in 889 patients. Figure 5 compares this number to the number of potentially eligible patients, enrolled patients, patients receiving the intervention from their GPs, as well as the number of patients that were treated with an intensity that would have made complete fidelity possible. Figure 5 indicates the steps that had to be accomplished to fulfil Fidelity criteria and shows that many patients were lost on the way.

### 2.4. Intervention Tailoring

**Figure 6:**
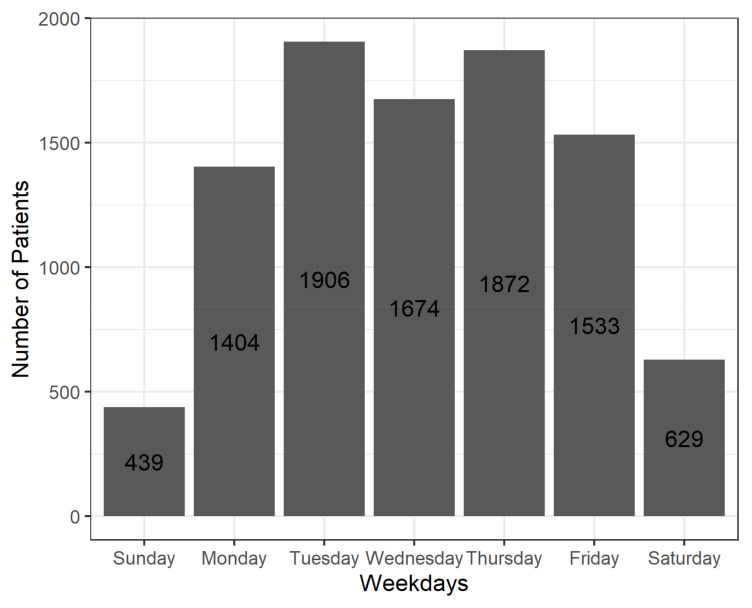
Number of patients, stratified by the day in the week when T_0_ was triggered.

**Figure 7:**
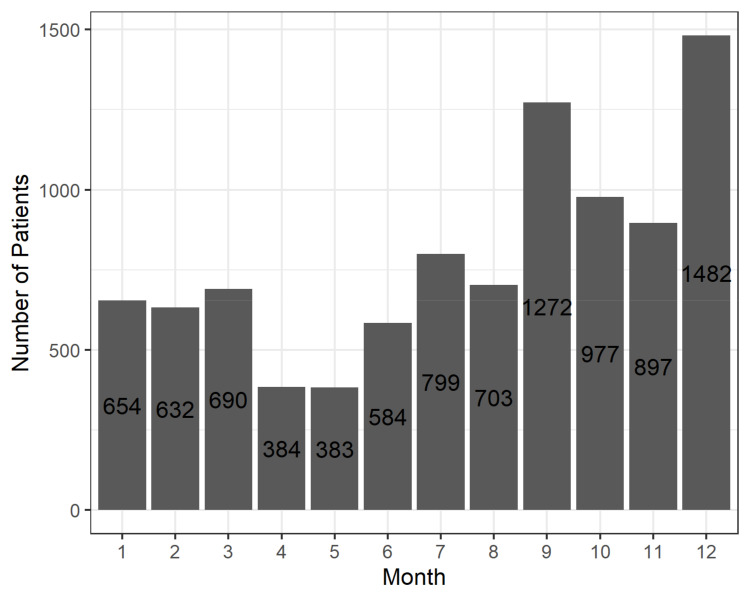
Number of patients stratified by month of the year when T_0_ was triggered.

**Figure 8:**
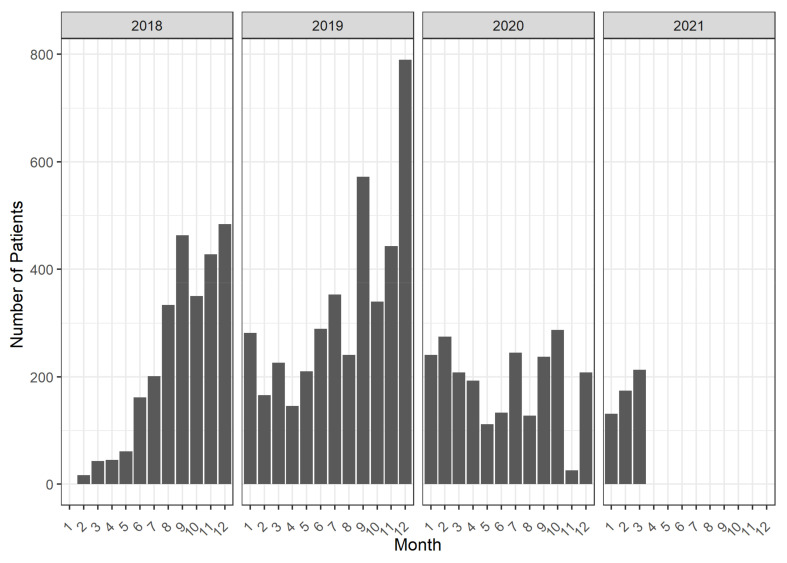
Number of patients, stratified by the month and year when T_0_ was triggered over the course of the study.

The “Tailoring” intervention dimension describes how participating GPs handled the intervention, and gives an indication how the intervention could be adapted to fit better into daily routines. Figure 6 shows on which days of the week T_0_ was triggered for patients, i.e., when the first GP assessment appeared in the software. In Figure 7, the same distribution is shown for the months of the year and in Figure 8, for the whole intervention period.

These analyses show when GPs preferred or had the chance to conduct medication reviews. The vast majority of cases (89%) were initiated between Monday and Friday, with peaks on Tuesdays and Thursdays. Only a small portion (11%) were initiated on Saturdays, Sundays, and public holidays, when practices are usually closed. In terms of months, the CDSS was used more often in the second half of the year, and especially in September and December. This pattern remains similar when the whole intervention period is examined, except for 2020, when the rise in patient cases was dampened by the COVID-19 pandemic.

Since the intervention software was in a test phase and on several occasions had to be updated, we initially planned to conduct sensitivity analyses to account for major software releases and lengthy software inaccessibility due to technical problems. However, as no error logbook was available, these analyses could not be conducted.

## 3. Discussion

### 3.1. Main Findings

Our process evaluation showed no relevant selection bias on either a patient, GP, or practice level in the participants included in the AdAM trial, compared to the eligible population in the study region (Intervention Reach). The reduction in the number of alerts was minimal (Intervention Dose) and all severe alerts were dealt with in only a modest number of participating patients, which was the final measure of a successful intervention (Intervention Fidelity). An analysis showed that the CDSS was most frequently used on days with long practice hours (Intervention Tailoring).

### 3.2. Our Findings in the Context of Existing Research

The inclusion criteria for our study were broad and the patient group correspondingly heterogeneous. Unlike other similar studies, the group was not preselected according to medication, disease, or age group [23,24]. As a result, the focus of GP training was on dealing with polypharmacy in general, and did not attempt to provide in-depth instruction on how to optimize specific cases, as is the case in polypharmacy trials with narrower inclusion criteria [25,26]. Moreover, some of the included patients did not profit from the intervention because there were very few or sometimes even no alerts that could be reacted to. This problem has also occurred in previous interventions [27].

Existing research into time-consuming documentation has shown that time efficiency is crucial for GPs, which was an implementation barrier in our study [28]. It is therefore plausible that a significant number of participating GPs failed to document all changes in the software. Previous studies have also indicated that integrating CDSS into the electronic health records of patients would improve the usefulness of medication reviews [23].

Furthermore, a recent systematic review found that physicians considered most alerts generated by CDSS to be unhelpful or inappropriate and therefore ignored them [29], which is confirmed by the low rate of documentation in eMMa. Moreover, a significant number of alerts required monitoring certain parameters such as blood potassium levels and cardiac rhythm, or changing drug intake schedules, at the same time as using the CDSS [30,31]. The software only registered the use of such strategies when the alert under consideration was marked as justified. The sensitivity analysis indicated that some GPs made use of this possibility, albeit only few, which was probably because the additional documentation time was not reflected in any improvement in the patient’s medication.

Difficulties in making medication changes arise when specialists are involved, as GPs are unwilling to interfere with their colleagues’ decisions [32], not least because patients like their specialists to be consulted before such decisions are made [33]. One solution may be to improve interdisciplinary cooperation so that complicated medication regimens, that require both time and practice, can be jointly assessed and improved. The involvement of pharmacists was not part of this intervention, but their support in conducting medication reviews would appear to be plausible, especially in view of the discussed implementation barriers, and existing literature, which indicates benefits in terms of both medication appropriateness [34] and patient-relevant outcomes such as quality of life [35]. Qualitative studies conducted in the AdAM trial suggest that the expertise of pharmacists is also appreciated by GPs [36] and patients [37]. In addition, as the AdAM intervention comprised only one voluntary two-hour education session, with accompanying online videos and FAQs, it is quite possible that better results could have been generated if training in polypharmacy and use of the software had been better, as can be seen in comparable trials [32,38,39].

### 3.3. Strengths and Limitations

The fact that the design of the AdAM study included an underlying process evaluation that had been planned and published beforehand, improves the methodological quality of the trial. Furthermore, this process evaluation addresses each step of the CDSS application process and responds to the urgent need for a deeper understanding of CDSS uptake reported in a recently published systematic review [40]. We could show that GPs attached more importance to severe alerts, and that alerts relating to medication dosage and kidney function were more frequently dealt with than those concerning e.g., drug–drug interactions or possible unsuitability due to a patient’s age (Intervention Dose). However, it should be taken into account that the software also generated alerts when a drug was entered into the system without additional information on the daily dosage or a patient’s renal function. The management of these alerts would not necessarily have resulted in any improvement in medication but simply have indicated that missing information had been entered into eMMa.

Our analyses also help understand the characteristics of participating GPs, and the kind of patients whose medication reviews they prioritized. Participating patients had a slightly lower average level of nursing care, indicating a barrier to the use of the intervention tools in nursing home patients and for home visits.

Overall, the documented changes were rather small, and all severe alerts were removed or justified in only few patients (Intervention Fidelity). However, it was not possible to distinguish between a patient’s medication having been left unchanged, or a change not having been documented in the software. As both the intended intensity of the intervention (Intervention Dose) and the desired intervention goal (Intervention Fidelity) were rarely fulfilled, conclusions about the potential risk reduction attributable to the intervention can only be drawn to a limited extent. Since the CDSS could not be linked with the practice management systems, GPs had to document all changes twice, which time constraints may have prevented, resulting in an underestimation of the use of eMMa.

Results in the Intervention Tailoring dimension showed that in the beginning of the intervention period, when updates and technical difficulties frequently occurred, the enrollment of patients in eMMa was low. GPs that were involved in the early stages of the intervention may have given up on the software after encountering technical problems early on. Furthermore, only few patients enrolled in the early months of each year, which coincided with the flu season. To the best of our knowledge, little research has been conducted into the impact of seasonal fluctuations on implementing a real-world study, so further analysis of the data gathered in the AdAM study may help in the planning of future interventions in clinical settings. Additionally, the COVID-19 pandemic struck during the intervention period. This unforeseeable event was a major, but certainly not the only disruption to the daily care of patients with polypharmacy that was observed during the course of this intervention, which makes the interpretation of results even more difficult.

### 3.4. Recommendations for Research and Clinical Practice

It is necessary to conduct more in-depth training before beginning such an intervention. An integration of the intervention tool in the practice management system and further measures to increase time efficiency would also facilitate adaptation and implementation for GPs and generate more robust data for scientific analysis. It is necessary to investigate whether the integration of further healthcare professionals, such as specialized physicians and pharmacists, would result in more effective medication reviews, especially in complex cases.

## 4. Materials and Methods

### 4.1. Background Information on the AdAM Study

The approach of the AdAM intervention (“*Anwendung für ein digital gestütztes Arzneimitteltherapie- und Versorgungsmanagement”*, or “application of digitally supported drug-therapy and care management”) is described in detail elsewhere [41]. In short, the intervention foresees that GPs perform at least one medication review in adult patients receiving five or more chronic medications with the help of a CDSS (software was developed under the name “eMMa”, which is an abbreviation for electronic medication management) that has been fed with all relevant medical information in the form of claims data from the statutory health insurance company BARMER. The primary aim is to decrease hospitalization and death rates among polypharmacy patients compared to a patient group receiving usual care.

### 4.2. eMMA

**Figure 9:**
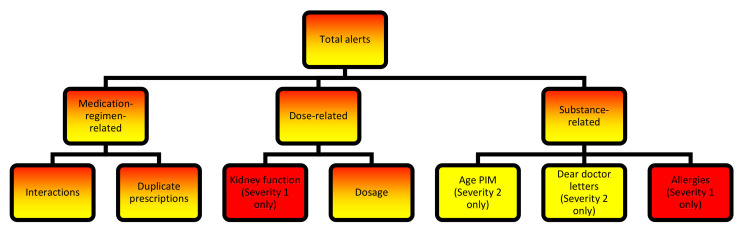
Alert categories documented by the software. Red font indicates alerts of the highest severity, and yellow font indicates medium severity.

**Figure 10:**
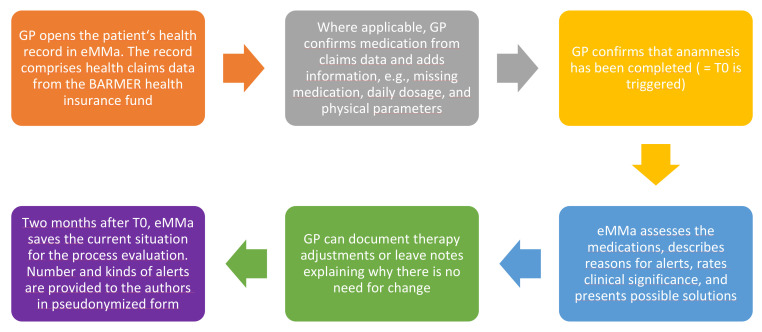
Schematic working process for GPs that use eMMa. The final step (purple field) defines T_1_.

The AdAM intervention involved the application of a CDSS that examined the medication of patients with polypharmacy after claims data provided by the patients’ statutory health insurance company had been entered into the system, and after GPs had confirmed the claims data and fed additional relevant information into the system themselves. The underlying software then generated alerts that were categorized by severity (of which only the two highest of four levels overall are analyzed here, since the two lower levels do not pose clinical significance) and type of potential inappropriateness. The alerts that could be seen by GPs and that were analyzed in this study are displayed in Figure 9. GPs then had the possibility to make medication changes and to discuss them with their patients. A detailed breakdown of the steps conducted by GPs is depicted in Figure 10, whereby both figures were previously published in our study protocol [21].

### 4.3. Theoretical Background of the Process Evaluation

The process evaluation is based on consensus recommendations in accordance with MRC guidance and the MRC process evaluation framework [18] and assesses four dimensions (Intervention Reach, Dose, Fidelity, Tailoring) of the implementation and application process. The defined dimensions and their adaptation to suit the implementation of the AdAM software are briefly explained below. A more detailed description can be found elsewhere [21].

### 4.4. Inclusion and Exclusion Criteria for Log Data Analysis

All patients to whom one of the following criteria applied, were included in the analysis of data extracted from the AdAM software:

1. The GP confirmed in the software that an anamnesis had been completed (referred to as “completed anamnesis”).
2. The software was used to print a medication plan.
3. At least five medications were entered into the software.

The inclusion criteria were prioritized in descending order: The first day on which criterion 1 was met was defined as T_0_. If this was never the case, criterion 2 and, if necessary, criterion 3 were treated analogously. Duplicates, i.e., patients whose pseudonym was included twice, were excluded after verification. In addition, patients were excluded if their GP only participated in the piloting test phase or had ceased to participate in the study before randomization (e.g., had retired). Patients that enrolled in eMMa after completion of the project were also excluded.

The Intervention Reach compares participants that fulfil the criteria to those that do not. All potentially eligible participants are therefore included in the analyses for that dimension.

### 4.5. Population and Outcomes

(a) Intervention Reach

This dimension deals with the “reach” of the intervention, i.e., whether the selection and inclusion of study participants was carried out as foreseen in the study protocol, and how the study population differed from the defined population in terms of the variables given in Appendix A, Table A1. These comparisons were conducted at the level of patients, physicians, and practices, and were used to determine structural similarity between the groups, and whether, for example, any particular group of patients was prioritized in the intervention.

For this purpose, all patients receiving the eMMa intervention (=active population, AP) were compared to:

- Study participants that had enrolled but did not receive the intervention (=inactive population, IP);
- Persons that fulfilled the entry criteria for the intention-to-treat population and were on the list of patients provided to participating GPs but did not take part in the intervention (=non-enrolled potentials, NEP).

Analogously, all GPs and practices enrolled in the AdAM study that cared for at least one AP patient, irrespective of whether they also treated patients in the IP or NEP groups were compared to:

- Enrolled GPs and practices that cared for at least one IP patient, but no AP patient;
- Enrolled GPs and practices that cared for at least one NEP patient, but no AP or IP patient.

**Figure 11:**
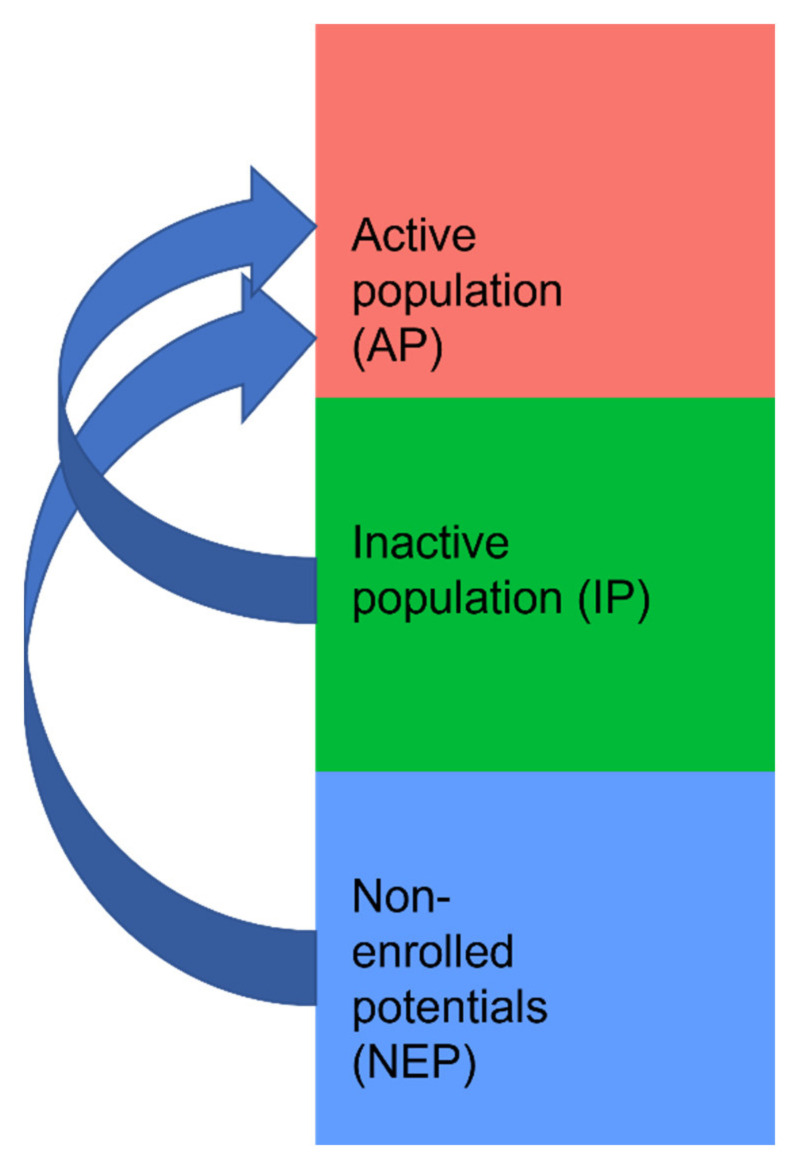
Populations that were compared for the Reach dimension.

An overview can be found in Figure 11.

Pseudonymized data for patient comparisons originated from BARMER’s data warehouse (a database in which all claims data are stored pseudonymously). Comparisons at GP and practice level were carried out using pseudonymized data from the association of statutory healthcare physicians in the study region (KVWL). Group comparisons were carried out using logistic regression and two-sided tests carried out with a significance level of alpha = 5%. Group membership was defined as the dependent variable.

(b) Intervention Dose

This dimension applies to the AP group only and assesses the extent of reductions in alerts in patients that received the AdAM intervention two months after patient data were originally entered into the software. In addition, prioritization associated with the severity and categories of alerts were also analyzed (Figure 9). The alerts were divided into justified (marked as processed or commented on by the GP) and unjustified alerts (not marked as processed or commented on by the GP). The number of alerts was measured at two points in time: the first timestamp (date) occurred when T_0_ is triggered, and automatically two months later (referred to as T_1_).

In this dimension, the main analysis is of the reduction in alerts between T_0_ and T_1_, stratified by severity and the category of alerts. In addition, sensitivity analyses were performed that only included unjustified alerts at T_1_.

Further sensitivity analyses only included the population for which GPs had confirmed that the anamnesis of the patient had been completed and that all medication been entered into the software. This was the original plan, whereby T_0_ was to be triggered in the software by pressing a button, and only then was it possible to deal with alerts. Before release, the software developers decided against making this process compulsory.

In order to adjust for clustering at a GP level, a multilevel Poisson model was calculated using the pseudonymized GP ID as a random effect. The total number of alerts was the dependent variable in the model, and T_0_ and T_1_ were the predictors. All models were stratified by age and sex.

(c) Intervention Fidelity

This dimension applies only to the AP group and examines the trustworthiness of the intervention, i.e., whether the software was used in such a way that a successful intervention (reduction in hospitalization and death) was possible. Alerts rated at the highest severity level were used to operationalize this dimension, as they were considered strongly indicative of a need for action. Furthermore, the GP had to have completed the anamnesis to show the software was being used as intended.

As long as all alerts at this level had been resolved or justified at T_1_, they were considered to have been successfully dealt with in terms of Fidelity.

For this dimension, we reported the proportion of patients whose serious warnings were completely resolved at T_1_. In addition, the analyses were stratified according to alert category.

(d) Intervention Tailoring

In contrast to the other dimensions, the focus here was on the individual adjustments GPs made in order to better integrate the intervention into daily practice routines. For this purpose, the temporal dimension of software use was investigated. Consequently, data are only analyzed for the AP group. Specifically, we looked at the number of patients whose data were called up for the first time on particular days of the week or in particular months, and looked for a concentration of such events during certain periods (e.g., at the weekend), or seasonal dependencies.

## 5. Conclusions

There are indications that the CDSS helped participating physicians prescribe fewer high-risk medications by encouraging them to adjust dosages, and to modify prescriptions to take account of renal function impairment. However, the intervention does not appear to have been used intensively, whereby it should be taken into consideration that utilization may have been under-reported in the log data. Overall, the results of the process evaluation indicate that the extent of the implementation of the AdAM intervention was weaker than anticipated.

## Figures and Tables

**Table 1:** Group comparison of AP and NEP patients.

Predictors	OR	95% CI	*p*-Value
(Intercept)	0.17	0.14–0.19	**<0.001**
**Age**	1.01	1.01–1.01	**<0.001**
**Sex** (ref: male)			
Female	0.93	0.89–0.98	**0.004**
**medCDS** *	1.04	0.98–1.09	0.177
**Nursing care level** (ref: 0)			
Nursing care level 1	0.76	0.65–0.88	**<0.001**
Nursing care level 2	0.74	0.68–0.80	**<0.001**
Nursing care level 3	0.52	0.46–0.57	**<0.001**
Nursing care level 4	0.34	0.29–0.40	**<0.001**
Nursing care level 5	0.27	0.21–0.35	**<0.001**
R^2^ Tjur	0.011		

* medCDS is a chronic disease score [22].

**Table 2:** Factors related to the chance of belonging to AP versus IP and AP versus NEP GPs.

	AP vs. IP			AP vs. NEP		
Predictors	OR	95% CI	*p*-Value	OR	95% CI	*p*-Value
(Intercept)	24.93	5.11–129.84	**<0.001**	5.31	1.97–14.51	**0.001**
**Specialization** (ref: Specialized in general practice)						
Without specialist qualification	0.48	0.18–1.35	0.145	1.05	0.45–2.42	0.909
Internist active in general practice	0.86	0.55–1.36	0.519	1.27	0.93–1.73	0.137
Other	0.34	0.03–7.45	0.384	1.1	0.10–23.83	0.94
**Age GP**	0.97	0.94–0.99	**0.018**	0.97	0.95–0.98	**<0.001**
**Sex GP** (ref: male)						
Female	1.03	0.65–1.63	0.906	1.02	0.75–1.38	0.9
**GP network** (ref: no)						
Yes	0.95	0.61–1.51	0.825	1.21	0.89–1.66	0.232
**Randomization group** (ref: control)						
Intervention	1.36	0.89–2.08	0.157	1.93	1.45–2.57	**<0.001**
R^2^ Tjur	0.023			0.051		

**Table 3:** Factors related to the chance of belonging to AP versus IP and AP versus NEP practices.

	AP vs. IP			AP vs. NEP		
Predictors	OR	95% CI	*p*-Value	OR	95% CI	*p*-Value
(Intercept)	2.41	1.05–5.52	**0.037**	1.02	0.61–1.72	0.933
**Number of GPs**	1.22	0.88–1.78	0.277	0.99	0.81–1.22	0.915
**Duration of practice**	1	0.98–1.03	0.735	0.99	0.97–1.01	0.278
**Type of practice** (ref: single practice)						
Group practice	0.95	0.48–1.87	0.878	1.75	1.10–2.79	**0.018**
Medical care center	-	-	0.987	1.12	0.15–9.86	0.913
**Randomization group** (ref: control)						
Intervention	1.34	0.82–2.18	0.24	2.02	1.45–2.82	**<0.001**
R^2^ Tjur	0.009			0.046		

**Table 4:** Overall number of alerts per category at T_0_ and change over the course of the study.

Analysis	Alert Category	Number of Alerts at T_0_ (Proportion of Total Alerts)	Change at T_1_ (%)	Number of Alerts of Severity 1 at T_0_ (Proportion of Total Alerts)	Change at T_1_ (%) (Severity 1 Alerts Only)
Main	Dosage	15,790 (56%)	−5.0	9530 (66%)	−6.2
	Kidney	2625 (9%)	−6.2	2625 (18%)	−6.2
	Interaction	5836 (21%)	−0.5	449 (3%)	−6.1
	Duplicate prescription	2323 (8%)	3.2	1615 (11%)	3.8
	Age	1653 (6%)	−0.1	N/A	N/A
	Allergy	143 (1%)	3.4	143 (1%)	3.4
Sensitivity “completed anamnesis”	Dosage	8879 (55%)	−9.6	5337 (66%)	−11.9
	Kidney	1489 (9%)	−11.5	1489 (18%)	−11.5
	Interaction	3400 (21%)	−1.6	250 (3%)	−11.6
	Duplicate prescription	1417 (9%)	3.2	990 (12%)	4.2
	Age	944 (6%)	−1.2	N/A	N/A
	Allergy	83 (1%)	3.4	83 (1%)	3.4

**Table 5:** Incidence rate ratio of alerts before and after the intervention, stratified by age, sex, alert category, and severity level.

		Incidence Rate Ratio (T_1_ vs. T_0_)					
Severity	Category	Female			Male		
		<65 Years	≥65 <85	≥85	<65 Years	≥65 <85	≥85
1 & 2	Dosage	0.95 (0.88–1.01)	0.94 (0.92–0.99) **	0.95 (0.90–1.00)	0.96 (0.89–1.03)	0.96 (0.91–1.00)	0.96 (0.88–1.06)
	Kidney	0.93 (0.77–1.13)	0.95 (0.87–1.04)	0.92 (0.81–1.03)	0.94 (0.76–1.14)	0.95 (0.85–1.07)	0.96 (0.78–1.18)
	Interaction	1.01 (0.90–1.13)	1.00 (0.94–1.06)	0.98 (0.89–1.09)	0.99 (0.88–1.12)	0.99 (0.92–1.07)	1.01 (0.87–1.18)
	Duplicate prescription	1.03 (0.90–1.19)	1.04 (0.94–1.15)	1.01 (0.84–1.21)	1.04 (0.88–1.22)	1.04 (0.92–1.17)	1.01 (0.78–1.41)
	Age	1.13 (0.67–1.72)	1.04 (0.99–1.06)	0.99 (0.84–1.15)	1.14 (0.56–1.87)	1.01 (0.88–1.17)	1.00 (0.74–1.43)
	Allergy	1.00 (0.55–1.83)	1.05 (0.74–1.50)	1.05 (0.59–1.85)	1.00 (0.42–2.40)	1.0 (0.57–1.76)	1.17 (0.39–3.47)
1	Dosage	0.93 (0.85–1.02)	0.94 (0.90–0.99) *	0.92 (0.86–0.99) *	0.95 (0.94–1.02)	0.95 (0.90–1.01)	0.94 (0.84–1.07)
	Kidney	0.93 (0.77–1.13)	0.95 (0.87–1.04)	0.92 (0.81–1.03)	0.94 (0.76–1.14)	0.95 (0.85–1.07)	0.96 (0.78–1.18)
	Interaction	0.94 (0.62–1.46)	0.93 (0.76–1.17)	0.93 (0.64–1.30)	0.96 (0.64–1.45)	0.93 (0.71–1.21)	1.04 (0.51–1.89)
	Duplicate prescription	1.04 (0.88–1.23)	1.05 (0.94–1.19)	1.01 (0.81–1.25)	1.05 (0.87–1.28)	1.03 (0.90–1.19)	1.01 (0.74–1.49)
	Allergy	1.00 (0.55–1.83)	1.05 (0.74–1.50)	1.05 (0.59–1.85)	1.00 (0.42–2.40)	1.00 (0.57–1.76)	1.23 (0.43–3.46)

** *p* < 0.01. * *p* < 0.05.

**Table 6:** Incidence rate ratio of unjustified alerts before and after the intervention, stratified by age, sex, alert category, and severity level.

Incidence Rate Ratio (T1 vs. T0)							
Severity	Category	Female			Male		
		<65 Years	≥65 <85	≥85	<65 Years	≥65 <85	≥85
1 & 2	Dosage	0.85 (0.79–0.91) ***	0.87 (0.84–0.90) ***	0.86 (0.82–0.91) ***	0.86 (0.80–0.93) ***	0.89 (0.85–0.93) ***	0.89 (0.81–0.98) *
	Kidney	0.78 (0.65–0.95) **	0.83 (0.76–0.95) ***	0.83 (0.73–0.94) **	0.83 (0.67–1.03)	0.86 (0.76–0.97) *	0.89 (0.72–1.10)
	Interaction	0.96 (0.86–1.08)	0.92 (0.87–0.98) **	0.90 (0.81–1.00)	0.93 (0.83–1.06)	0.93 (0.86–1.00)	0.93 (0.80–1.09)
	Duplicate prescription	1.00 (0.87–1.14)	1.01 (0.91–1.11)	0.94 (0.79–1.14)	1.02 (0.86–1.20)	1.01 (0.89–1.15)	0.97 (0.74–1.27)
	Age	1.01 (0.78–1.46)	0.95 (0.85–1.02)	0.92 (0.79–1.07)	1.13 (0.59–1.90)	0.94 (0.82–1.11)	0.91 (0.67–1.23)
	Allergy	1.00 (0.55–1.83)	1.02 (0.71–1.46)	0.96 (0.53–1.82)	1.00 (0.42–2.40)	0.92 (0.51–1.63)	1.17 (0.39–3.47)
1	Dosage	0.81 (0.77–0.82) ***	0.85 (0.81–0.89) ***	0.83 (0.77–0.89) ***	0.84 (0.81–0.86) ***	0.87 (0.82–0.93) ***	0.87 (0.82–0.93) ***
	Kidney	0.783 (0.64–0.95) **	0.83 (0.76–0.91) ***	0.83 (0.73–0.94) **	0.83 (0.67–1.03)	0.86 (0.76–0.97) *	0.89 (0.72–1.10)
	Interaction	0.86 (0.55–1.34)	0.85 (0.67–1.07)	0.84 (0.60–1.19)	0.80 (0.52–1.24)	0.79 (0.60–1.04)	0.92 (0.53–1.61)
	Duplicate prescription	1.00 (0.85–1.19)	1.02 (0.91–1.15)	0.94 (0.76–1.18)	1.03 (0.85–1.25)	1.02 (0.88–1.17)	0.99 (0.73–1.36)
	Allergy	0.96 (0.53–1.72)	1.02 (0.71–1.46)	0.96 (0.53–1.72)	1.00 (0.42–2.40)	0.92 (0.51–1.63)	1.17 (0.39–3.47)

*** *p* < 0,001, ** *p* < 0,01, * *p* < 0,05.

**Table 7:** Proportion of patients fulfilling the Fidelity criteria.

Alert Category	Number of Patients for Whom the Number of Severe Alerts is >0 at T_0_	Number of Patients Fulfilling Fidelity Criteria	Proportion of Patients Fulfilling Fidelity Criteria
Dosage	3210	780	24.3%
Kidney	1322	383	29.0%
Interaction	246	71	28.9%
Duplicate prescription	787	57	7.2%
Allergy	64	4	6.2%

## Data Availability

The data presented in this study are available on request from the corresponding author. The data are not publicly available due to data protection regulations.

## Appendix A

**Table A1:** Variables for the Reach dimension (ref = reference category in logistic regression).

Comparison 1	Comparison 2	Patients	GPs		Practices	
				Factor Levels		Factor Levels
**AP versus IP**	**AP versus NEP**	**Age (years)**	**Age** (years)		**Type of practice**	
						*Single practice (ref)*
						*Group practice*
						*Medical care center*
		**Sex**	**Sex**		**Number of employed GPs**	
				*Male (ref)*	**Time since practice inception (years)**	
				*Female*	**Randomization group**	
		**medCDS**	**Specialization type**			*Intervention*
		**Nursing care level care** (0 (ref), 1–5)		*Specialized in general practice (ref)*		*Control (ref)*
				*GP without specialization*		
				*Internist active in general practice*		
				*Other*		
			**Participation in a GP network**			
				*Yes*		
				*No (ref)*		
			**Randomization group**			
				*Intervention*		
				*Control (ref)*		
